# Daily Stress and Sleep Associations Vary by Work Schedule: A Between- and Within-Person Analysis in Nurses

**DOI:** 10.1093/geroni/igab046.433

**Published:** 2021-12-17

**Authors:** Danica Slavish, Jessica Dietch, Heidi Kane, Joshua Wiley, Yang Yap, Kimberly Kelly, Camilo Ruggero, Daniel Taylor

**Affiliations:** 1 University of North Texas, Denton, Texas, United States; 2 Oregon State University, Corvallis, Oregon, United States; 3 University of Texas at Dallas, Richardson, Texas, United States; 4 Monash University, Clayton, Victoria, Australia; 5 University of Arizona, Tucson, Arizona, United States

## Abstract

Nurses experience poor sleep and high stress due to demanding work environments. Night shift work may exacerbate stress-sleep associations. We examined bidirectional associations between daily stress and sleep, and moderation by shift worker status and daily work schedule. 392 nurses (92% female, mean age = 39.54) completed 14 days of sleep diaries and actigraphy, plus daily assessments of stress and work schedule upon awakening. Nurses were classified as recent night shift workers if they worked 1+ night during the past 14 days. Greater daily stress predicted shorter diary sleep duration and lower diary sleep efficiency. Shorter diary and actigraphy sleep duration and lower diary sleep efficiency predicted higher next-day stress. Compared to recent night workers, day workers had higher stress after nights with shorter sleep. Associations did not vary by daily work schedule. Sleep disturbances and stress may unfold in a toxic cycle and are prime intervention targets among nurses.

